# Supplementary material for: Using a real-world network to model the trade-off between stay-at-home restriction, vaccination, social distancing and working hours on COVID-19 dynamics
Source: PeerJ. 2022 Dec 15;10:e14353. doi: 10.7717/peerj.14353 (PMC9760027; doi:10.7717/peerj.14353)
Supplement: Figure S1 — Let Y represent encounters which occurred between 6m and 20m distance in Halsemere data point set, and X represent total number of logged data points of each Y during three consecutive day. The duration between 07:00 AM up to 08:30 AM and 18:00 PM up to 23:00 PM on Thursday and Friday represent by t1. The duration between 07:00 AM up to 23:00 PM on Saturday represent by t2. The duration between 08:30 AM up to 18:00 PM on Thursday and Friday represent by t3. Let Z(t1+ t2) give the total number Y which occurred in t1 and t2 duration, and Z(t3) give the total number Y which occurred in t3. [file peerj-10-14353-s001.pdf]

```
if X ≤ 80
    if X > 15
        if Z(t1 + t2) > Z(t3)
            Y classified as household contact
        else
            Y classified as workplace contact
        endif
    else
        if Z(t1 + t2) > Z(t3)
            Y classified as social environment contact
        else
            Y classified as workplace contact
        endif
    endif
endif
```
