# Supplementary figures and images for: Using a real-world network to model the trade-off between stay-at-home restriction, vaccination, social distancing and working hours on COVID-19 dynamics
Source: PeerJ. 2022 Dec 15;10:e14353. doi: 10.7717/peerj.14353 (PMC9760027; doi:10.7717/peerj.14353)

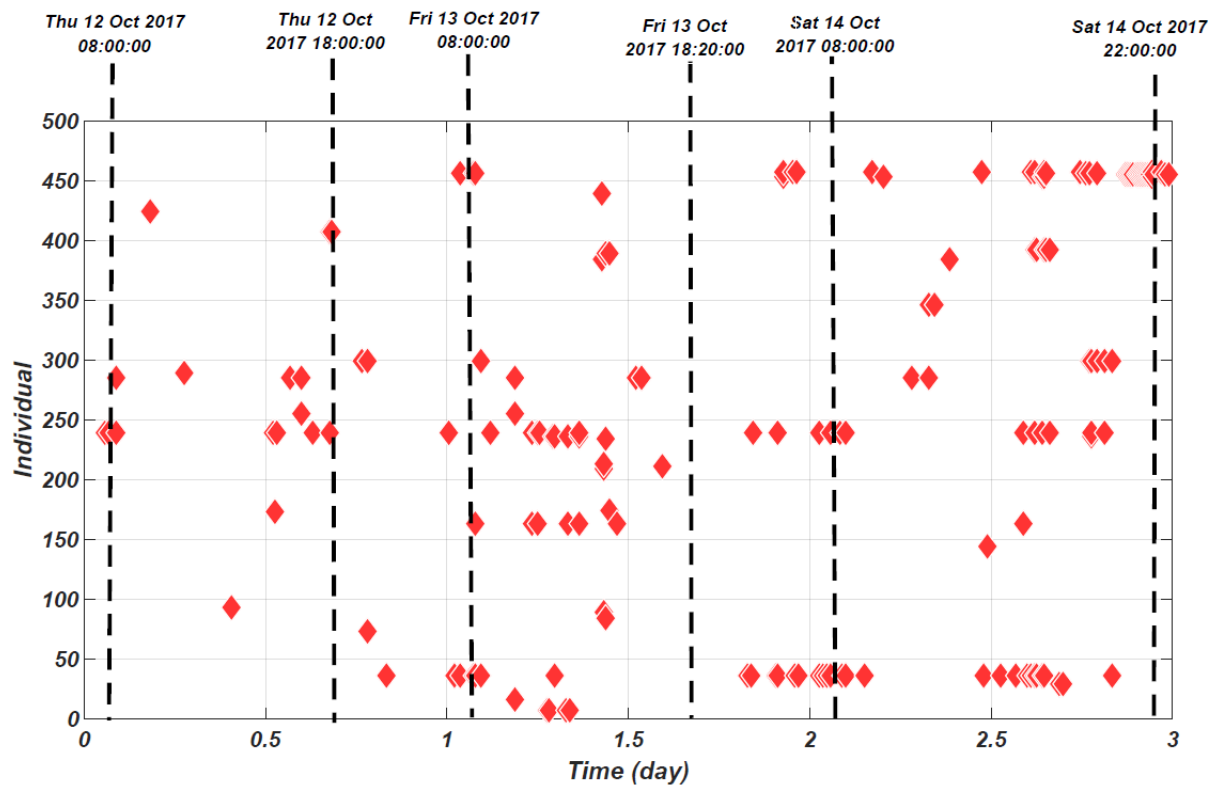

Supplement: Figure S2 — Demonstrates the behavior and contact member of every single individual Haslemere data set during these three consecutive days.In here the behavior of 21st individual during three consecutive days. The red diamond shapes represent every single individual. The black dash line illustrates specific hours during these three days [file peerj-10-14353-s002.pdf]

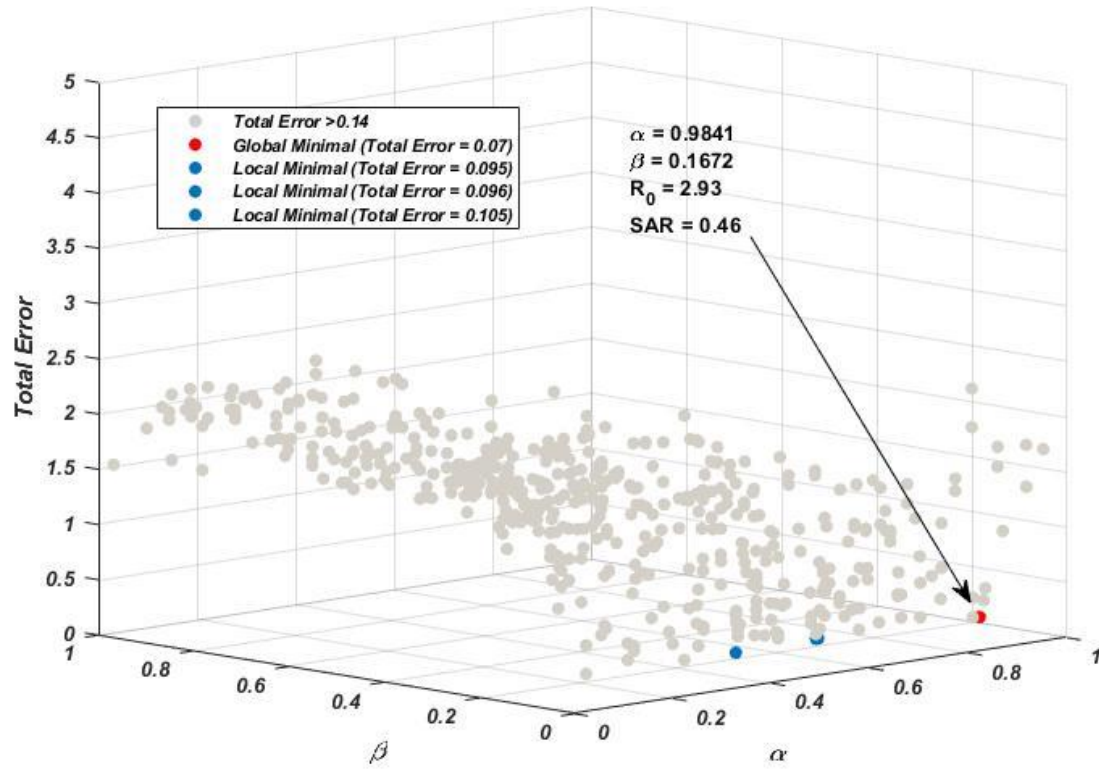

Supplement: Figure S4 — For calculating scaling parameters of distance (α) and transmission rate (β). [file peerj-10-14353-s004.pdf]

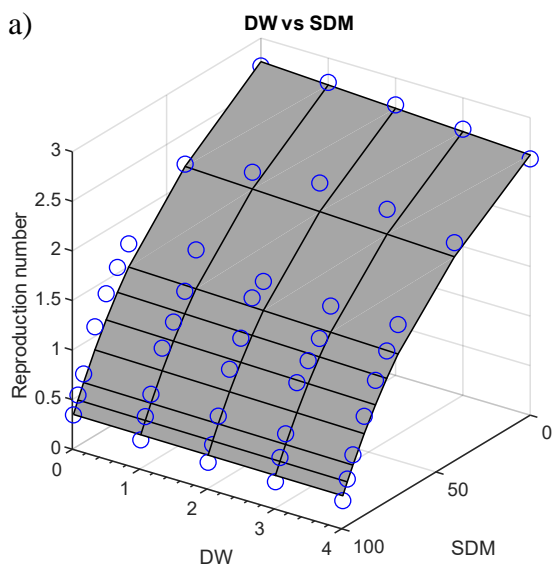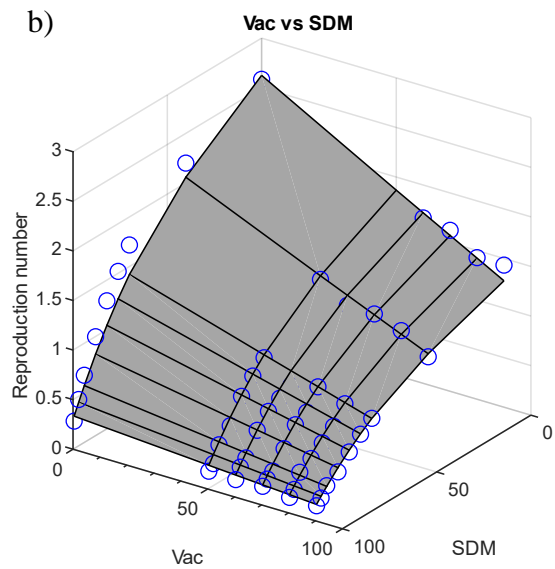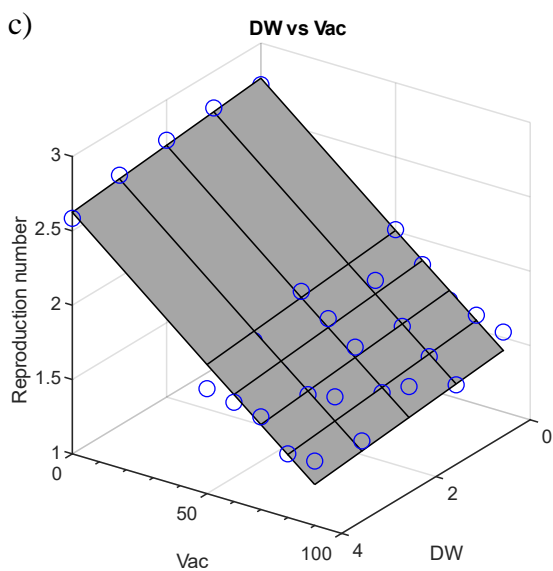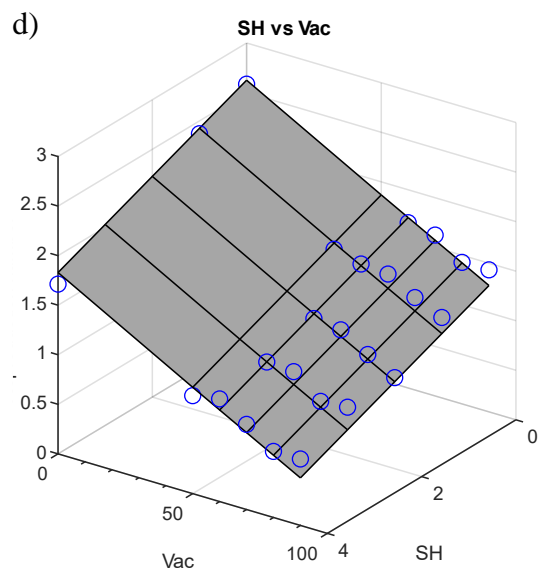

Supplement: Figure S5 — (A) When the decrease in working hours (DW) is plotted against the social distancing measure (SDM). (B) when the vaccination percentage (Vac) is plotted against the social distancing measure (SDM). (C) when the decrease in working hours (DW) is plotted against vaccination percentage (Vac). (D) When stay-at-home restriction (SH) is plotted against vaccination percentage (Vac) [file peerj-10-14353-s005.pdf]

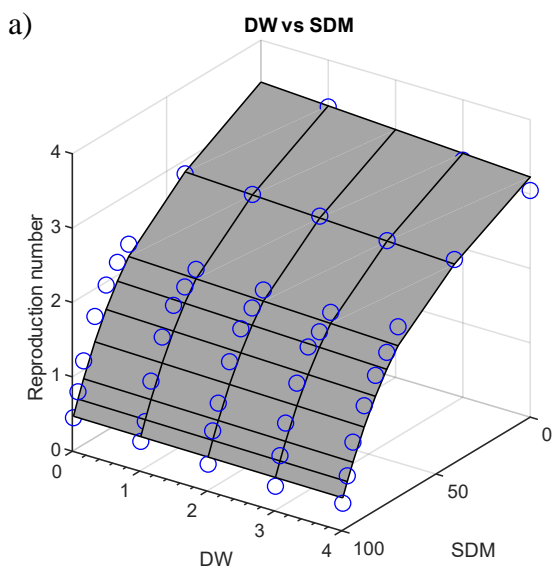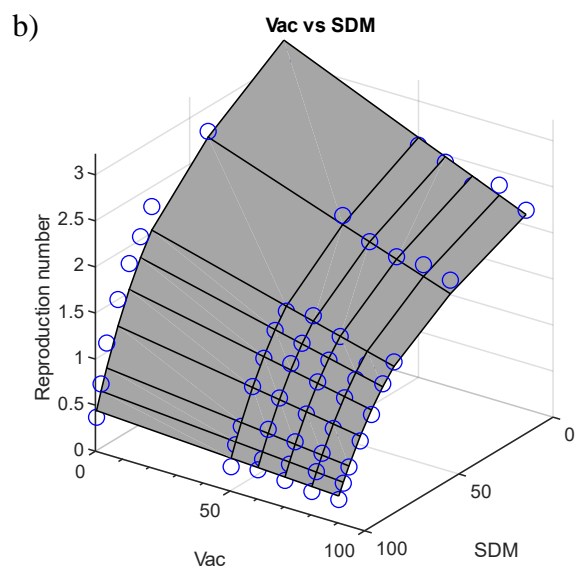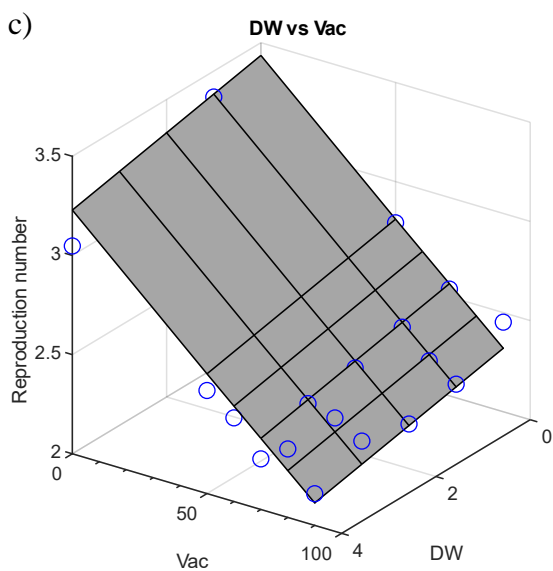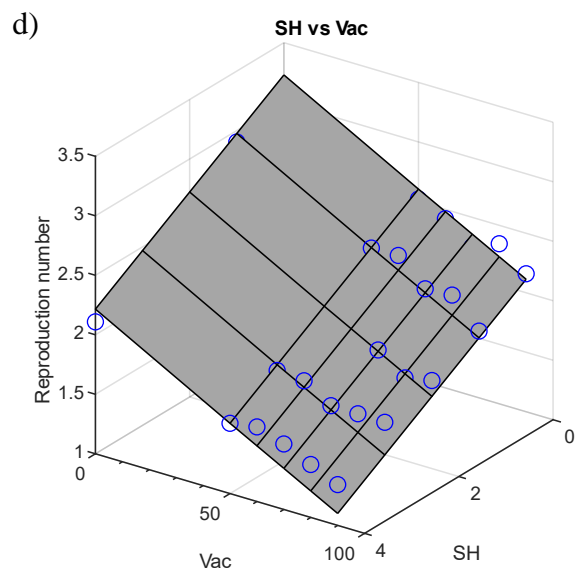

Supplement: Figure S6 — (A) When the decrease in working hours (DW) is plotted against the social distancing measure (SDM). (B) when the vaccination percentage (Vac) is plotted against the social distancing measure (SDM). (C) when the decrease in working hours (DW) is plotted against vaccination percentage (Vac). (D) When stay-at-home restriction (SH) is plotted against vaccination percentage (Vac). [file peerj-10-14353-s006.pdf]
