# Supplementary material for: Using a real-world network to model the trade-off between stay-at-home restriction, vaccination, social distancing and working hours on COVID-19 dynamics
Source: PeerJ. 2022 Dec 15;10:e14353. doi: 10.7717/peerj.14353 (PMC9760027; doi:10.7717/peerj.14353)
Supplement: Figure S3 — In this figure, the first two diagonal cells show the number and percentage of correct classifications by the algorithm. For example, 110 contacts are correctly classified as household. This corresponds to 8.15% of all 1895 contacts. Similarly, 492 contacts are correctly classified as workplace. This corresponds to 36.44% of all contacts.in same way, 642 contacts are correctly classified as social environment. This corresponds to 47.56% of all contacts. 1 of the household contacts is incorrectly classified as workplace contact and this corresponds to 0.07% of all 1895 contacts. Also, 12 of the household contacts is incorrectly classified as social environment contact and this corresponds to 0.89% of all 1,895 contacts. Similarly, 4 of the workplace contacts are incorrectly classified as households and this corresponds to 0.3% of all data. In same way, 18 of the workplace contacts are incorrectly classified as social environment and this corresponds to 1.33% of all data. 26 of the social environment contacts are incorrectly classified as households and this corresponds to 1.93% of all data. In same way, 45 of the social environment contacts are incorrectly classified as workplace and this corresponds to 3.33% of all data. Out of 123 household predictions, 78.57% are correct and 21.43% are wrong. Out of 514 workplace predictions, 91.45% are correct and 8.55% are wrong. Out of 713 social environment predictions, 95.54% and 4.46% are wrong. Out of 52 household contacts, 89.43% are correctly classified as household and 10.57% are classified as workplace and social environment. Out of 590 workplace contacts, 95.72% are correctly classified as household and 4.28% are classified as household and social environment. Out of 1003 social environment contacts, 90.04% are correctly classified as household and 9.96% are classified as workplace and households. Overall, 91.73% of the predictions are correct and 8.27% are wrong. [file peerj-10-14353-s003.pdf]

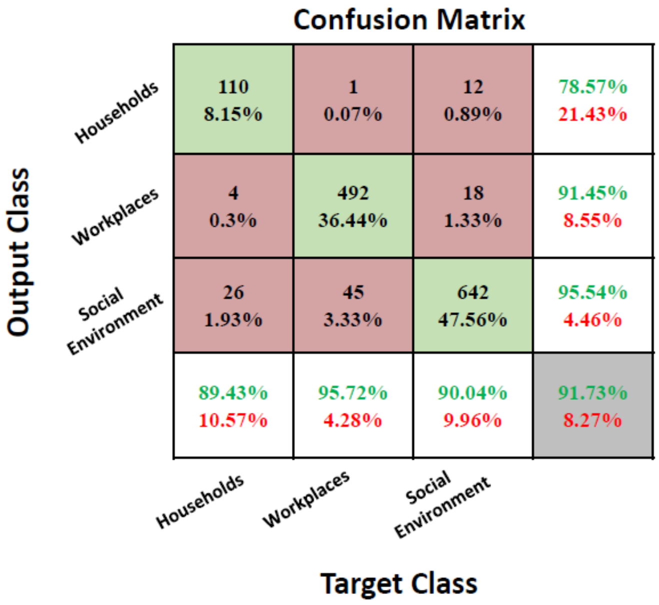
Confusion Matrix
Households
Workplaces
Social Environment
Output Class
110
8.15%
1
0.07%
12
0.89%
78.57%
21.43%
4
0.3%
492
36.44%
18
1.33%
91.45%
8.55%
26
1.93%
45
3.33%
642
47.56%
95.54%
4.46%
89.43%
10.57%
95.72%
4.28%
90.04%
9.96%
91.73%
8.27%
Households
Workplaces
Social Environment
Target Class
