# Supplementary material for: Using a real-world network to model the trade-off between stay-at-home restriction, vaccination, social distancing and working hours on COVID-19 dynamics
Source: PeerJ. 2022 Dec 15;10:e14353. doi: 10.7717/peerj.14353 (PMC9760027; doi:10.7717/peerj.14353)
Supplement: Table S1 — Demonstrates the behavior and contact member of every single individual of Haslemere data set during these three consecutive days [file peerj-10-14353-s007.docx]

**Table S1:**

**List of procedures that are obtained by using visualization method.**

Demonstrates the behavior and contact member of every single individual of Haslemere data set during these three consecutive days

| **Categories** | **Procedures** |
| --- | --- |
| Household | 1. Individuals with at least 10 logged data points between 22:00 and 07:55 AM on any of the dates [1] 2. Individuals who contact for three consecutive night after 19:00 and have to distance less than 2 3. Individuals who have more than 80 logged data point during three consecutive day |
| Workplace | 1. Encounters which occurred on Thursday and Friday between 8:30 AM and 18:00 |
| Social Environment | 1. Individuals with a lease than ten logged data points between 22:00 and 07:55 AM on any of the dates 2. The encounters which are occurred after 19:00 clock for one-night lease than five logged data point 3. Encounters which occurred on Saturday from 8:00 AM and 18:00 less 15 logged data points |

1] Stephen M. Kissler, Petra Klepac, Maria Tang, Andrew J.K. Conlan and Julia R. Gog, ” Supplemental Information for: Sparking “The BBC Four Pandemic”: Leveraging citizen science and mobile phones to model the spread of disease, April 7, 2019
