# Supplementary material for: Using a real-world network to model the trade-off between stay-at-home restriction, vaccination, social distancing and working hours on COVID-19 dynamics
Source: PeerJ. 2022 Dec 15;10:e14353. doi: 10.7717/peerj.14353 (PMC9760027; doi:10.7717/peerj.14353)
Supplement: Table S2 [file peerj-10-14353-s008.docx]

**Table S2:**

**Agent based model parameters and their values**

| **Parameter** | **Definition** | **Value** | **Source** |
| --- | --- | --- | --- |
| $d_{1}$ | mean number of days in the latency stage | 2.7 day | Derived by finding the difference between incubation period and pre-symptomatic period. We take incubation period 5.1 day [7] |
| $d_{2}$ | mean number of days in pre-symptomatic stage | 2.4 day | [2] |
| $d_{3}$ | mean number of days in the asymptomatic stage | 5.4 day | Assumed to be the same duration as the total infectious period for symptomatic cases, including pre-symptomatic transmission [3] |
| $\boldsymbol{s}$ | the ratio of symptomatic cases | 0.83 | [4] |
| $d_{4}$ | average time of going to the hospital | 3 day | [5] |
| $\alpha$ | Infection Probability constant | $0.9841$ | Derived (Fitted to COVID-19 $R_{0}$ and SAR of household by sampling (see SI Fig. 2)) |
| $\beta$ | Infection Probability constant | 0.1672 | Derived (Fitted to COVID-19 $R_{0}$ and SAR of household by sampling (see SI Fig. 2)) |
| $\mu_{H}$ | Transmission reduction fator for asymptomatic cases inside household | 0.696 | [6] |
| $\mu_{O}$ | Transmission reduction fator for asymptomatic cases outside household | 0.42 | [6] |
| $g$ | granularity | 192 | Derived by converting 1 day to 5 minutes intervals (16×12) |

[2] Zhongxing Ding, Kai Wang, Mingwang Shen, Kai Wang, Shi Zhao, Wenyu Song, Rui Li, Estimating the time interval between transmission generations and the presymptomatic period by contact tracing surveillance data from 31 provinces in the mainland of China, Fundamental Research, Volume 1, Issue 2, 2021, Pages 104-110, ISSN 2667-3258, https://doi.org/10.1016/j.fmre.2021.02.002.

[3] Nicholas G Davies, Adam J Kucharski, Rosalind M Eggo, Amy Gimma, W John Edmunds, Thibaut Jombart, Effects of non-pharmaceutical interventions on COVID-19 cases, deaths, and demand for hospital services in the UK: a modelling study, The Lancet Public Health, Volume 5, Issue 7, 2020, Pages e375-e385, ISSN 2468-2667, https://doi.org/10.1016/S2468-2667(20)30133-X.

[4] Oyungerel Byambasuren, Magnolia Cardona, Katy Bell, Justin Clark, Mary-Louise McLaws, Paul Glasziou, Estimating the extent of asymptomatic COVID-19 and its potential for community transmission: systematic review and meta-analysis, medRxiv 2020.05.10.20097543; doi: https://doi.org/10.1101/2020.05.10.20097543

[5] Faes C, Abrams S, Van Beckhoven D, et al. Time between Symptom Onset, Hospitalisation and Recovery or Death: Statistical Analysis of Belgian COVID-19 Patients. Int J Environ Res Public Health. 2020;17(20):7560. Published 2020 Oct 17. doi:10.3390/ijerph17207560

[6] Qifang Bi, Justin Lessler, Isabella Eckerle, Stephen A Lauer, Laurent Kaiser, Nicolas Vuilleumier, Derek AT Cummings, Household Transmission of SARS-CoV-2: Insights from a Population-based Serological Survey, medRxiv 2020.11.04.20225573; doi: <https://doi.org/10.1101/2020.11.04.20225573>

[8] Stephen A. Lauer, Kyra H. Grantz, Qifang Bi, Forrest K. Jones. The Incubation Period of Coronavirus Disease 2019 (COVID-19) From Publicly Reported Confirmed Cases: Estimation and Application, Annals of Internal Medicine May 5, 2020 172(9):577, https://www.acpjournals.org/doi/full/10.7326/M20-0504
