# Supplementary material for: Using a real-world network to model the trade-off between stay-at-home restriction, vaccination, social distancing and working hours on COVID-19 dynamics
Source: PeerJ. 2022 Dec 15;10:e14353. doi: 10.7717/peerj.14353 (PMC9760027; doi:10.7717/peerj.14353)
Supplement: Table S3 [file peerj-10-14353-s009.docx]

**Table S3**

**List of Scenarios and number of simulations that are made by using agent-based model.**

| **Figure** | **Scenario** | **Number of simulations** |
| --- | --- | --- |
| 2a | Estimating effective reproduction number (*R_e_*) number for transmission reduction that conducted by fixing household transmission rate (*β_h_*) and varying non-household Transmission rate (*β_o_*) using real network. | 500 |
|  | Estimating *R_e_* for transmission reduction that conducted by varying both *β_h_* and *β_o_* using real network. | 500 |
|  | Estimating *R_e_* for transmission reduction that conducted by varying both *β_h_* and *β_o_* using random network. | 500 |
|  | Estimating *R_e_* for transmission reduction that conducted by fixing household transmission rate (*β_h_*) and varying non-household Transmission rate (*β_o_*) using random network. | 500 |
| 2b | Finding infection occurrence ratio for *R_e_* in the household, workplaces, and social environment when *β_h_* fix and *β_o_* varies. | 500 |
| 2c | Finding infection occurrence ratio for R_e_ in the household, workplaces, and social environment when *β_h_* and *β_o_* varies. | 500 |
| 3 | Estimating third order transmission chain occurrence ratio for R_0_ = 2.87 using real network | 500 |
|  | Estimating third order transmission chain occurrence ratio for R_0_ = 2.87 using random network | 500 |
|  | Estimating third order transmission chain occurrence ratio for R_0_ = 1 using random network | 500 |
|  | Estimating third order transmission chain occurrence ratio for R_0_ = 1 using real network by varying both *β_h_* and *β_o_* | 500 |
|  | Estimating third order transmission chain occurrence ratio for R_0_ = 1 using real network by fixing *β_h_* and conducting 88% reduction in *β_o_* | 500 |
| 5a | Estimating basic reproduction number R_0_ for free weekend without stay-at-home restriction | 500 |
|  | Estimating basic reproduction number R_0_ for stay-at-home restriction on Sunday | 500 |

**Table S3**

**List of Scenarios and number of simulations that are made by using agent-based model (Continue).**

|  | Estimating basic reproduction number R_0_ for stay-at-home restriction on Sunday and Saturday | 500 |
| --- | --- | --- |
| 5b | Finding infection occurrence ratio for *R_0_* in the household, workplaces, and social environment when there is no stay-at-home | 500 |
| 5c | Finding infection occurrence ratio for *R_0_* in the household, workplaces, and social environment when there is stay-at-home restriction on Sunday | 500 |
| 5d | Finding infection occurrence ratio for *R_0_* in the household, workplaces, and social environment when there is stay-at-home restriction on Sunday and Saturday | 500 |
| 7a | Estimating basic reproduction number for wild-type COVID-19 (R0_WT_) for:  1) 0%,50%,60%,70%,80%,90% vaccination of population (Vac= [0,50,60,70,80,90])  2) 0,1,2,3,4 hour(s) decrease in working hour (DW= [0,1,2,3,4])  3) 0.1,2,3,4 day(s) stay-at-home restriction (SH= [0,1,2,3,4])  4) 0%,40%,70%,76%,82%,88%,94%,97%,99% social distancing measures (SDM= [0,40,70,76,82,88,94,97,99]) | 500* |
| 7b | Estimating basic reproduction number for delta variant COVID-19 (R0_delta_) for:  1) 0%,50%,60%,70%,80%,90% vaccination of population (Vac= [0,50,60,70,80,90])  2) 0,1,2,3,4 hour(s) decrease in working hour (DW= [0,1,2,3,4])  3) 0.1,2,3,4 day(s) stay-at-home restriction (SH= [0,1,2,3,4])  4) 0%,40%,70%,76%,82%,88%,94%,97%,99% social distancing measures (SDM= [0,40,70,76,82,88,94,97,99]) | 500** |

***** We run 500 simulations for each Vac, DW, SH, and SDM level, exclusively. For instance, estimating R0_WT_ for Vac=0, DW=0, SH=0, and SDM= 0 accepted as one scenario. Consequently, there are 1350 different scenarios.

****** We run 500 simulations for each Vac, DW, SH, and SDM level, exclusively. For instance, estimating R0_delta_ for Vac=0, DW=0, SH=0, and SDM= 0 accepted as one scenario. Consequently, there are 1350 different scenarios.
