# Supplementary material for: Using a real-world network to model the trade-off between stay-at-home restriction, vaccination, social distancing and working hours on COVID-19 dynamics
Source: PeerJ. 2022 Dec 15;10:e14353. doi: 10.7717/peerj.14353 (PMC9760027; doi:10.7717/peerj.14353)
Supplement: Table S4 — Here, the root means square errors (RMSD) are calculated to find the best mathematical expression to the simulated estimates of wild-type COVID-19 reproduction number (R0WT). The equations have four parameters: Decrease in working hours DW, social distancing measure (SDM), stay-at-home restriction (SH), and vaccination ratio (Vac). The row shaded by grey demonstrates the best mathematical expression for simulated data. [file peerj-10-14353-s010.docx]

**Table S4:**

**Fitting multidimensional surfaces to simulated data of the agent-based model.**

Here, the root means square errors (RMSD) are calculated to find the best mathematical expression to the simulated estimates of wild-type COVID-19 reproduction number (R0_WT_). The equations have four parameters: Decrease in working hours DW, social distancing measure (SDM), stay-at-home restriction (SH), and vaccination ratio (Vac). The row shaded by grey demonstrates the best mathematical expression for simulated data.

|  | **Equation** | **RMSD** |
| --- | --- | --- |
| A | $d+a\cdot DW+b\cdot SH+c\cdot\frac{\mathrm{Vac}}{100}+e\cdot\frac{\mathrm{SDM}}{100}$ | 0.0303 |
| B | $d+a\cdot DW+b\cdot SH+c\cdot\frac{\mathrm{Vac}}{100}+e\cdot\frac{\mathrm{SDM}}{100}+f\cdot\left( \frac{\mathrm{SDM}}{100} \right)^{2}$ | 0.0248 |
| C | $d+a\cdot DW+b\cdot SH+c\cdot\frac{\mathrm{Vac}}{100}+f\cdot\left( \frac{\mathrm{Vac}}{100} \right)^{2}+e\cdot\frac{\mathrm{SDM}}{100}$ | 0.0293 |
| D | $d+a\cdot DW+b\cdot SH+f\cdot\left( \mathrm{SH} \right)^{2}+c\cdot\frac{\mathrm{Vac}}{100}+e\cdot\frac{\mathrm{SDM}}{100}$ | 0.0302 |
| E | $d+a\cdot DW+f\cdot\left( \mathrm{DW} \right)^{2}+b\cdot SH+c\cdot\frac{\mathrm{Vac}}{100}+e\cdot\frac{\mathrm{SDM}}{100}$ | 0.0303 |
| F | $\left( d+a\cdot DW+b\cdot SH+c\cdot\frac{\mathrm{Vac}}{100} \right)\cdot\left( 1+e\cdot\frac{\mathrm{SDM}}{100} \right)$ | 0.0139 |
| G | $\left( d+a\cdot DW+b\cdot SH+c\cdot\frac{\mathrm{SDM}}{100} \right)\cdot\left( 1+e\cdot\frac{\mathrm{Vac}}{100} \right)$ | 0.0196 |
| H | $\left( d+a\cdot DW+b\cdot\frac{\mathrm{Vac}}{100}+c\cdot\frac{\mathrm{SDM}}{100} \right)\cdot\left( 1+e\cdot SH \right)$ | 0.0224 |
| J | $\left( d+a\cdot SH+b\cdot\frac{\mathrm{Vac}}{100}+c\cdot\frac{\mathrm{SDM}}{100} \right)\cdot\left( 1+e\cdot DW \right)$ | 0.0300 |
| K | $\left( d+a\cdot DW \right)\cdot\left( 1+b\cdot SH \right)\cdot\left( 1+c\cdot\frac{\mathrm{Vac}}{100} \right)\cdot\left( 1+e\cdot\frac{\mathrm{SDM}}{100} \right)$ | 0.0131 |
| L | $\left( d+a\cdot DW+b\cdot SH \right)\cdot\left( 1+c\cdot\frac{\mathrm{Vac}}{100} \right)\cdot\left( 1+e\cdot\frac{\mathrm{SDM}}{100} \right)$ | 0.0130 |
| M | $\left( d+a\cdot DW+b\cdot SH+c\cdot\frac{\mathrm{Vac}}{100} \right)\cdot\left( 1+e\cdot\frac{\mathrm{SDM}}{100}+f\cdot\left( \frac{\mathrm{SDM}}{100} \right)^{2} \right)$ | 0.0060 |
| N | $\left( d+a\cdot DW+b\cdot SH+c\cdot\frac{\mathrm{Vac}}{100}+f\cdot\left( \frac{\mathrm{Vac}}{100} \right)^{2} \right)\cdot\left( 1+e\cdot\frac{\mathrm{SDM}}{100} \right)$ | 0.0130 |

**Table S4:**

**Fitting multidimensional surfaces to simulated data of the agent-based model (Continue).**

Here, the root means square errors (RMSD) are calculated to find the best mathematical expression to the simulated estimates of wild-type COVID-19 reproduction number (R0_WT_). The equations have four parameters: Decrease in working hours DW, social distancing measure (SDM), stay-at-home restriction (SH), and vaccination ratio (Vac). The row shaded by grey demonstrates the best mathematical expression for simulated data.

| O | $\left( d+a\cdot DW+b\cdot SH+f\cdot\left( \mathrm{SH} \right)^{2}+c\cdot\frac{\mathrm{Vac}}{100} \right)\cdot\left( 1+e\cdot\frac{\mathrm{SDM}}{100} \right)$ | 0.0139 |
| --- | --- | --- |
| P | $\left( d+a\cdot DW+f\cdot\left( \mathrm{DW} \right)^{2}+b\cdot SH+c\cdot\frac{\mathrm{Vac}}{100} \right)\cdot\left( 1+e\cdot\frac{\mathrm{SDM}}{100} \right)$ | 0.0139 |
| R | $\left( d+a\cdot DW+b\cdot SH+c\cdot\frac{\mathrm{Vac}}{100}+e\cdot\frac{\mathrm{SDM}}{100}+f\cdot\left( \frac{\mathrm{SDM}}{100} \right)^{2}+g\cdot\left( \frac{\mathrm{SDM}}{100} \right)^{3} \right)$ | 0.0246 |
| S | $\left( d+a\cdot DW+b\cdot SH+c\cdot\frac{\mathrm{Vac}}{100} \right)\cdot\left( e\cdot\frac{\mathrm{SDM}}{100}+f\cdot\left( \frac{\mathrm{SDM}}{100} \right)^{2}+g\cdot\left( \frac{\mathrm{SDM}}{100} \right)^{3} \right)$ | 0.0057 |
