# Supplementary material for: Using a real-world network to model the trade-off between stay-at-home restriction, vaccination, social distancing and working hours on COVID-19 dynamics
Source: PeerJ. 2022 Dec 15;10:e14353. doi: 10.7717/peerj.14353 (PMC9760027; doi:10.7717/peerj.14353)
Supplement: Table S5 — Here, the root means square errors (RMSD) are calculated to find the best mathematical expression to the simulated estimates of delta variant COVID-19 reproduction number (R0delta). The equations have four parameters: Decrease in working hours DW, social distancing measure (SDM), stay-at-home restriction (SH), and vaccination ratio (Vac). The row that is shaded by grey demonstrates the best mathematical expression for simulated data. [file peerj-10-14353-s011.docx]

**Table S5:**

**Fitting multidimensional surfaces to simulated data of the agent-based model.**

Here, the root means square errors (RMSD) are calculated to find the best mathematical expression to the simulated estimates of delta variant COVID-19 reproduction number (R0_delta_). The equations have four parameters: Decrease in working hours DW, social distancing measure (SDM), stay-at-home restriction (SH), and vaccination ratio (Vac). The row that is shaded by grey demonstrates the best mathematical expression for simulated data.

|  | **Equation** | **RMSD** |
| --- | --- | --- |
| A | $d+a\cdot DW+b\cdot SH+c\cdot\frac{\mathrm{Vac}}{100}+e\cdot\frac{\mathrm{SDM}}{100}$ | 0.0534 |
| B | $d+a\cdot DW+b\cdot SH+c\cdot\frac{\mathrm{Vac}}{100}+e\cdot\frac{\mathrm{SDM}}{100}+f\cdot\left( \frac{\mathrm{SDM}}{100} \right)^{2}$ | 0.0253 |
| C | $d+a\cdot DW+b\cdot SH+c\cdot\frac{\mathrm{Vac}}{100}+f\cdot\left( \frac{\mathrm{Vac}}{100} \right)^{2}+e\cdot\frac{\mathrm{SDM}}{100}$ | 0.0530 |
| D | $d+a\cdot DW+b\cdot SH+f\cdot\left( \mathrm{SH} \right)^{2}+c\cdot\frac{\mathrm{Vac}}{100}+e\cdot\frac{\mathrm{SDM}}{100}$ | 0.0534 |
| E | $d+a\cdot DW+f\cdot\left( \mathrm{DW} \right)^{2}+b\cdot SH+c\cdot\frac{\mathrm{Vac}}{100}+e\cdot\frac{\mathrm{SDM}}{100}$ | 0.0534 |
| F | $\left( d+a\cdot DW+b\cdot SH+c\cdot\frac{\mathrm{Vac}}{100} \right)\cdot\left( 1+e\cdot\frac{\mathrm{SDM}}{100} \right)$ | 0.0437 |
| G | $\left( d+a\cdot DW+b\cdot SH+c\cdot\frac{\mathrm{SDM}}{100} \right)\cdot\left( 1+e\cdot\frac{\mathrm{Vac}}{100} \right)$ | 0.0498 |
| H | $\left( d+a\cdot DW+b\cdot\frac{\mathrm{Vac}}{100}+c\cdot\frac{\mathrm{SDM}}{100} \right)\cdot\left( 1+e\cdot SH \right)$ | 0.0465 |
| J | $\left( d+a\cdot SH+b\cdot\frac{\mathrm{Vac}}{100}+c\cdot\frac{\mathrm{SDM}}{100} \right)\cdot\left( 1+e\cdot DW \right)$ | 0.0529 |
| K | $\left( d+a\cdot DW \right)\cdot\left( 1+b\cdot SH \right)\cdot\left( 1+c\cdot\frac{\mathrm{Vac}}{100} \right)\cdot\left( 1+e\cdot\frac{\mathrm{SDM}}{100} \right)$ | 0.0435 |
| L | $\left( d+a\cdot DW+b\cdot SH \right)\cdot\left( 1+c\cdot\frac{\mathrm{Vac}}{100} \right)\cdot\left( 1+e\cdot\frac{\mathrm{SDM}}{100} \right)$ | 0.0434 |
| M | $\left( d+a\cdot DW+b\cdot SH+c\cdot\frac{\mathrm{Vac}}{100} \right)\cdot\left( 1+e\cdot\frac{\mathrm{SDM}}{100}+f\cdot\left( \frac{\mathrm{SDM}}{100} \right)^{2} \right)$ | 0.0111 |
| N | $\left( d+a\cdot DW+b\cdot SH+c\cdot\frac{\mathrm{Vac}}{100}+f\cdot\left( \frac{\mathrm{Vac}}{100} \right)^{2} \right)\cdot\left( 1+e\cdot\frac{\mathrm{SDM}}{100} \right)$ | 0.0438 |

**Table S5:**

**Fitting multidimensional surfaces to simulated data of the agent-based model (Continue).**

Here, the root means square errors (RMSD) are calculated to find the best mathematical expression to the simulated estimates of delta variant COVID-19 reproduction number (R0_delta_). The equations have four parameters: Decrease in working hours DW, social distancing measure (SDM), stay-at-home restriction (SH), and vaccination ratio (Vac). The row that is shaded by grey demonstrates the best mathematical expression for simulated data.

| O | $\left( d+a\cdot DW+b\cdot SH+f\cdot\left( \mathrm{SH} \right)^{2}+c\cdot\frac{\mathrm{Vac}}{100} \right)\cdot\left( 1+e\cdot\frac{\mathrm{SDM}}{100} \right)$ | 0.0437 |
| --- | --- | --- |
| P | $\left( d+a\cdot DW+f\cdot\left( \mathrm{DW} \right)^{2}+b\cdot SH+c\cdot\frac{\mathrm{Vac}}{100} \right)\cdot\left( 1+e\cdot\frac{\mathrm{SDM}}{100} \right)$ | 0.0437 |
| R | $\left( d+a\cdot DW+b\cdot SH+c\cdot\frac{\mathrm{Vac}}{100}+e\cdot\frac{\mathrm{SDM}}{100}+f\cdot\left( \frac{\mathrm{SDM}}{100} \right)^{2}+g\cdot\left( \frac{\mathrm{SDM}}{100} \right)^{3} \right)$ | 0.0223 |
| S | $\left( d+a\cdot DW+b\cdot SH+c\cdot\frac{\mathrm{Vac}}{100} \right)\cdot\left( e\cdot\frac{\mathrm{SDM}}{100}+f\cdot\left( \frac{\mathrm{SDM}}{100} \right)^{2}+g\cdot\left( \frac{\mathrm{SDM}}{100} \right)^{3} \right)$ | 0.0072 |
